# Role of digital health in improving physical and mental well-being during COVID-19 pandemic

**DOI:** 10.1192/j.eurpsy.2021.740

**Published:** 2021-08-13

**Authors:** F. Arain, A. Tohid, H. Arain, S. Afzal, M.S. Tauheed, M. Tauheed, M. Zeshan, W. Azeem

**Affiliations:** 1 Psychiatry, BronxCare Health System Mount Sinai, NY, United States of America; 2 Psychiatry, University of Southern California, Los Angeles, United States of America; 3 Under Graduate, Columbia University, New York, United States of America; 4 Psychiatry, Ziauddin University Hospital, Karachi, Pakistan; 5 Internal Medicine, Dow University of Health Science, Karachi, Pakistan; 6 Psychiatry, Rutgers New Jersey Medical School Newark, New Jersey, United States of America; 7 Psychiatry, Weill Cornell Medical College, New York, United States of America

**Keywords:** Digital Health, Tele-mental health, COVID-19, Pandemic

## Abstract

**Introduction:**

Due to the COVID-19 pandemic, there is a steep rise in the acceptance of telemedicine and digital health, including increased interest in pursuing mental health treatment through telepsychiatry. Digital health helps following social distancing measures and increases the health outcomes.

**Objectives:**

To see the role of digital health in improving physical and mental well-being during COVID-19 Pandemic

**Methods:**

This study is a part of a large global project where 240 people inquired advice on phone app during COVID-19-Pandemic. Later on, a short study was conducted on the same population through survey to evaluate the effectiveness of digital health/tele-mental health. We also searched PubMed, Google Scholar, PsychInfo, and Medline for words “Digital Health, Tele-mental health, COVID-19-Pandemic”. Reviewed 40 articles and included 3 in this review^1,4,5^.

**Results:**

We received a total of 98 responses. 65.6% people reported that online health resources are helpful in relieving pandemic-induced anxiety/stress, 66.2% reported to continue online health services after pandemic, 37.7% noted that digital health saves times in waiting areas, 46% reported lack of physical interaction with doctor as a disadvantage of digital health, and 40.3% reported comfort in using tele-mental health. Our literature review has shown barriers like privacy concerns and technological issues^1^. Provision of tele-psychiatry is safe and effective in continuity of mental health care.^4,5^

**Conclusions:**

There has been an increased inclination towards digital health during any disaster. During COVID-19-Pandemic, digital health has increased access to mental health care and reduced risk of infection. The drawbacks include poor patient-doctor relationship, reimbursement concerns, and lack of confidentiality.

